# Methods for Identifying *Neisseria meningitidis* Carriers: A Multi-Center Study in the African Meningitis Belt 

**DOI:** 10.1371/journal.pone.0078336

**Published:** 2013-10-23

**Authors:** Nicole E. Basta, James M. Stuart, Maria C. Nascimento, Olivier Manigart, Caroline Trotter, Musa Hassan-King, Daniel Chandramohan, Samba O. Sow, Abdoulaye Berthe, Ahmed Bedru, Yenenesh K. Tekletsion, Jean-Marc Collard, Jean-François Jusot, Aldiouma Diallo, Hubert Basséne, Doumagoum M. Daugla, Khadidja Gamougam, Abraham Hodgson, Abudulai A. Forgor, Babatunji A. Omotara, Galadima B. Gadzama, Eleanor R. Watkins, Lisa S. Rebbetts, Kanny Diallo, Noel S. Weiss, M. Elizabeth Halloran, Martin C. J. Maiden, Brian Greenwood

**Affiliations:** 1 Department of Ecology and Evolutionary Biology, Princeton University, Princeton, New Jersey, United States of America; 2 Research and Policy for Infectious Disease Dynamics, Fogarty International Center, National Institutes of Health, Bethesda, Maryland, United States of America; 3 Vaccine and Infectious Disease Division, Fred Hutchinson Cancer Research Center, Seattle, Washington, United States of America; 4 Faculty of Infectious and Tropical Diseases, Department of Disease Control, London School of Hygiene & Tropical Medicine, London, United Kingdom; 5 Department of Veterinary Medicine, University of Cambridge, Cambridge, United Kingdom; 6 Centre pour le Développement des Vaccins, Bamako, Mali; 7 Armauer Hansen Research Institute, Addis Ababa, Ethiopia; 8 Department of Zoology, University of Oxford, Oxford, United Kingdom; 9 Centre de Recherche Médicale et Sanitaire, Yantala, Niamey, Niger; 10 Instiutut de Recherche pour le Développement, Dakar, Senegal; 11 Centre de Support en Santé Internationale, N’Djamena, Chad; 12 Research and Development Division, Ghana Health Service, Accra, Ghana; 13 War Memorial Hospital, Navrongo, Ghana; 14 Department of Community Medicine, University of Maiduguri, Maiduguri, Borno State, Nigeria; 15 Department of Medical Microbiology, University of Maiduguri, Maiduguri, Borno State, Nigeria; 16 Department of Epidemiology, School of Public Health, University of Washington, Seattle, Washington, United States of America; 17 Department of Biostatistics, School of Public Health, University of Washington, Seattle, Washington, United States of America; Pennsylvania State University College of Medicine, United States of America

## Abstract

**Objective:**

Detection of meningococcal carriers is key to understanding the epidemiology of *Neisseria meningitidis*, yet no gold standard has been established. Here, we directly compare two methods for collecting pharyngeal swabs to identify meningococcal carriers.

**Methods:**

We conducted cross-sectional surveys of schoolchildren at multiple sites in Africa to compare swabbing the posterior pharynx behind the uvula (U) to swabbing the posterior pharynx behind the uvula plus one tonsil (T). Swabs were cultured immediately and analyzed using molecular methods.

**Results:**

One thousand and six paired swab samples collected from schoolchildren in four countries were analyzed. Prevalence of meningococcal carriage was 6.9% (95% CI: 5.4-8.6%) based on the results from both swabs, but the observed prevalence was lower based on one swab type alone. Prevalence based on the T swab or the U swab alone was similar (5.2% (95% CI: 3.8-6.7%) versus 4.9% (95% CI: 3.6-6.4%) respectively (p=0.6)). The concordance between the two methods was 96.3% and the *kappa* was 0.61 (95% CI: 0.50-0.73), indicating good agreement.

**Conclusions:**

These two commonly used methods for collecting pharyngeal swabs provide consistent estimates of the prevalence of carriage, but both methods misclassified carriers to some degree, leading to underestimates of the prevalence.

## Introduction

Devastating, large-scale meningitis outbreaks have occurred in the African meningitis belt every 5-12 years for the past century, resulting in an annual incidence as high as 1,000 cases per 100,000 population during epidemics [1-3]. Typical annual incidence in non-epidemic periods ranges from 1 to 20 cases per 100,000 population [2]. Asymptomatic carriers of *Neisseria meningitidis* serve as a reservoir for persistence and spread of the bacterium in the population. Understanding the epidemiology and natural history of carriers is central to understanding the epidemiology of meningococcal disease. Interest in this area has increased in recent years, particularly in the context of understanding the effects of conjugate vaccines on carriage [4-8]. In the African meningitis belt, where efforts to prevent major meningococcal epidemics caused by serogroup A meningococci have intensified with the introduction of a new serogroup A polysaccharide-protein conjugate vaccine (PsA-TT MenAfriVac^TM^) [9-13], further investigation of the natural history of carriage, better methods for conducting surveillance of carriage, and a clearer understanding of the relationship between carriage, outbreaks of invasive disease, and immunity is needed [14]. 

While sample collection methods for identifying cases of invasive meningococcal disease from cerebrospinal fluid are well established, questions remain about the most effective method for collecting pharyngeal swabs to identify asymptomatic carriers. Methods used in previous studies have not been consistent, with variation in both the region of the pharynx sampled and the treatment of the swab immediately following collection [5,6,15,16]. A recent review compared common collection methods and found that collecting the swab through the mouth rather than through the nose, touching the swab to the posterior pharynx wall alone instead of the tonsils alone, and plating the sample immediately rather than using transport medium improved the identification of carriers [17]. However, the authors of the review argued that further evidence is needed to directly assess whether there is a difference between collecting the sample by swabbing the posterior pharynx alone or in combination with swabbing the tonsils. No previous study has directly addressed this question. In addition, the ecology of pharyngeal carriage of meningococcal and other bacteria is not well understood. Whether swabbing the tonsils and the posterior pharynx would improve the detection of carriers by sampling two surfaces or whether other bacteria living on the tonsils might inhibit growth of the meningococcus is not known. 

Evaluating methods for collecting swabs to identify *N. meningitidis* carriage is key to identification of carriers, to reducing misclassification, and to implementing large-scale studies of carriage, including appropriately powered longitudinal studies. The African Meningococcal Carriage Consortium (MenAfriCar, www.menafricar.org) is an international collaboration that was established in 2009 to define the epidemiology of meningococcal carriage across the meningitis belt before and after the introduction of PsA-TT vaccine [18]. Prior to the implementation of large, cross-sectional carriage surveys, a multi-center pilot study was conducted in schoolchildren to compare the two pharyngeal swabbing methods for detecting carriage of *N. meningitidis* described above. 

## Materials and Methods

### Ethics Statement

The study was approved by the Ethics Committee at the London School of Hygiene & Tropical Medicine and the relevant ethical committees at each of the seven participating African centers (*Comité National d'Ethique pour la Recherche en Santé* (*CNERS*) in Senegal; Research & Ethical Committee of the University of Maiduguri Teaching Hospital in Nigeria; *Comité Consultatif National d'Ethique* in Niger; *Comité d'Ethique de la Faculté de Médecine et Pharmacie D’Odonto-Stomatologie de l’Université de Bamako* in Mali; Navrongo Health Research Center Institutional Review Board in Ghana; Armauer Hansen Research Institute/All Africa Leprosy Rehabilitation and Training Center (AHRI/ALERT) Ethical Review Committee in Ethiopia, and in Chad approval was granted by a committee established to oversee MenAfriCar studies by the Ministry of Health since no formal ethical committee was in place in the country at the time). During enrollment, staff explained the purpose and nature of the study, and a parent or guardian provided written, informed consent. Older children provided written assent (age determined by local practices) and younger children provided oral assent.

During 2009 and early 2010, cross-sectional surveys were conducted in schoolchildren at five urban sites (N'Djamena, Chad; Navrongo, Ghana; Butajira, Ethiopia; Bamako, Mali; Maiduguri, Nigeria;) and two rural sites (Say, Niger; Niakhar, Senegal) in seven countries of the African meningitis belt to compare methods for collecting pharyngeal swabs. Standard field and laboratory operating procedures were implemented in all centers; these have been described previously [18]. The samples collected in Chad, Ghana, and Nigeria had to be discarded following technical difficulties maintaining proper storage temperatures; results from the surveys in Ethiopia, Mali, Niger, and Senegal are reported in this analysis.

Each center chose one or more schools in an area where no meningitis vaccination campaign had occurred in the previous two years (or previous six months in Niger) and recruited a convenience sample until the target sample size of 250 children in each center was reached. Children were eligible if they were 5–15 years of age, had no severe acute or long-term illness, and if they had not received a meningitis vaccine during the specified time period. 

Two pharyngeal samples were collected from each child using sterile, dacron-tipped swabs with plastic shafts. Two techniques for swabbing the pharynx through the mouth were compared; one method involved swabbing the posterior pharynx behind the uvula (hereafter referred to as method “U”) and the other method involved swabbing both the posterior pharynx behind the uvula and one tonsillar fossa (hereafter referred to as method “T”). The order of the swabbing method was alternated every 25 participants in Ethiopia, and every 20 participants in Mali; in Niger and Senegal the U swab was collected followed by the T swab throughout. The sample labels did not indicate which method was used for sample collection so that the laboratory personnel could not distinguish between the T and U swabs during processing. 

Modified Thayer-Martin (TM) selective agar plates were prepare locally using Gonococci agar base (Oxoid CM0367B), hemoglobin powder (Oxoid LP053B) containing 3mg/liter vancomycin, 7.5mg/liter colistin, 12.5 U/liter nystatin, 5mg/liter trimetropin lactate (Oxoid SR00991E) and Vitox enrichment supplement (Oxoid SR0090A) (Thermo Scientific, UK) [19]. Swabs were plated immediately in the field onto TM plates, returned to the laboratory within six hours, and incubated in 5% CO_2_ at 35-37°C for 24-48 hours to determine growth. A single colony with morphology typical of *N. meningitidis* (large or medium size, blue-grey color, and mucoid in appearance) was selected, sub-cultured on a blood agar plate (BAP), streaked, and incubated in 5% CO_2_ at 35-37°C for an additional 18-24 hours. BAPs were prepared locally with blood agar base number 2 (Oxoid, CM0271, Thermo Scientific, UK) supplemented with 5% defibrinated sheep’s blood. The colonies remaining on the TM selective agar plate were collected with a sterile plastic loop, suspended in a cryotube containing 1mL of Brain heart infusion (BHI) broth supplemented with 15% glycerol and stored at -80°C. The remaining colonies from the BAP were emulsified in 0.5 mL phosphate buffered saline (PBS) in microcentrifuge tubes, boiled for 20 minutes to release DNA, cooled, divided into four aliquots in 250μL tubes and stored at -20°C for future molecular testing. 

During the pilot survey in Mali, the selected colonies sub-cultured onto BAP underwent oxidase testing and Gram staining to ensure that they were Gram-negative diplococci. However, DNA samples prepared from the selected colonies sent from Mali to the University of Oxford for molecular testing showed that these samples contained DNA from many organisms that were not Gram-negative diplococci. This prompted the inclusion of three additional biochemical tests in a new protocol circulated to all the sites: γ-glutamyl transferase activity (GGT) (Rosco Diagnostica, Denmark) for identification of presumptive *N. meningitidis*, β-galactosidase activity with ortho-nitrophenyl-*β*-D-galactopyranoside (ONPG) (Rosco Diagnostica, Denmark) for identification of *Neisseria lactamica*, and butyrate esterase activity (Tributyrin) (Rosco Diagnostica, Denmark) to further distinguish *Moraxella* species from *Neisseria* species which was the main cause of the initial misidentification [18]. The new protocol piloted in Mali in June 2010 indicated that the introduction of the biochemical tests improved species identification. The new protocol incorporating the biochemical tests was introduced in the rest of the sites two months later using samples from the original pilot study that had been stored in BHI broth supplemented with glycerol at -80°C. These samples were thawed at room temperature, vortex mixed briefly, and plated on TM plates followed by BAPs as described above. Growth from all oxidase positive, Gram negative diplococcic samples were harvested into microcentrifuge tubes containing 1 mL PBS, placed in a boiling water bath for 20 minutes to release DNA and inactivate nucleases, cooled, divided into lots and stored at -20°C for molecular testing. Heat killed cell suspensions, prepared from all oxidase-positive, Gram-negative diplococci from each site (49 from Ethiopia, 188 from Mali, 23 from Niger, 95 from Senegal) were sent to the University of Oxford for molecular characterization [18].

A swab was positive for *N. meningitidis* if the *rplF* sequence-based assay, described previously in [18], identified *N. meningitidis*. A participant was classified as a positive carrier if at least one of the samples provided met this definition. Data for each center were collected and managed locally using Microsoft Excel; data were cleaned and merged centrally using STATA for Mac version 12.1 (StataCorp LP 2012). Data were analyzed to determine the overall prevalence of *N. meningitidis* among the swabs collected and among the participants overall and at each center using standard statistical measures and calculating the exact binomial 95% confidence intervals (CIs) (also known as the Clopper-Pearson CIs) [20] for the point prevalence estimates. The two methods for collecting pharyngeal swabs were compared by calculating the concordance, *kappa*, and applying McNemar’s test for paired samples to test the null hypothesis that there was no difference between the proportion of positive samples observed using one method compared to the other. Analyses were performed combined (pooling all samples) and by center. Data were analyzed using STATA for Mac version 12.1 (StataCorp LP 2012).

## Results

From the 1013 children enrolled across the four centers included in this analysis, a total of 2023 T or U pharyngeal swabs were obtained. There was minor variation in the distribution of participants by age and gender across the study sites (Table 1). Five percent of the swabs collected (95% CI: 4.1-6.0%) were positive for *N. meningitidis*. Two U swabs were collected from five children and two other children had missing information on the swab type; these were excluded from the analysis. Paired T and U samples from 1006 children were analyzed; 6.9% (95% CI: 5.4-8.6%) of children were positive for *N. meningitidis* carriage by at least one swabbing method. Prevalence of carriage by age group (50.7% of carriers were aged 5-10 years old, 46.4% were aged 11-15 years old) and sex (49.3% of carriers were female) were similar. 

**Table 1:** Summary of characteristics of the samples collected overall and by center.

	***Ethiopia***	***Mali***	***Niger***	***Senegal***	***Total***
*Number of Schools*	*2*	*1*	*5*	*7*	*15*
*Dates of Survey*	*12/2009-1/2010*	*6/2009*	*11/2009*	*10-11/2009*	*-*
*Age*					
*5-10 years*	*102*	*122*	*157*	*145*	*526*
*11-15 years*	*148*	*128*	*102*	*105*	*483*
*Sex*					
*Male*	*125*	*125*	*113*	*117*	*480*
*Female*	*125*	*125*	*150*	*133*	*533*
*Swabbing Method Alternated*	*Per 25 children*	*Per 20 children*	*-*	*-*	*-*
*Participants Enrolled*	*250*	*250*	*263*	*250*	*1013*
*T Swabs Collected* ^+^	*248*	*250*	*262*	*246*	*1006*
*U Swabs Collected* ^+^	*249*	*250*	*264*	*254*	*1017*

+ *Three swabs from two children were missing their T or U status and were excluded from this analysis.*

**Figure 1:**
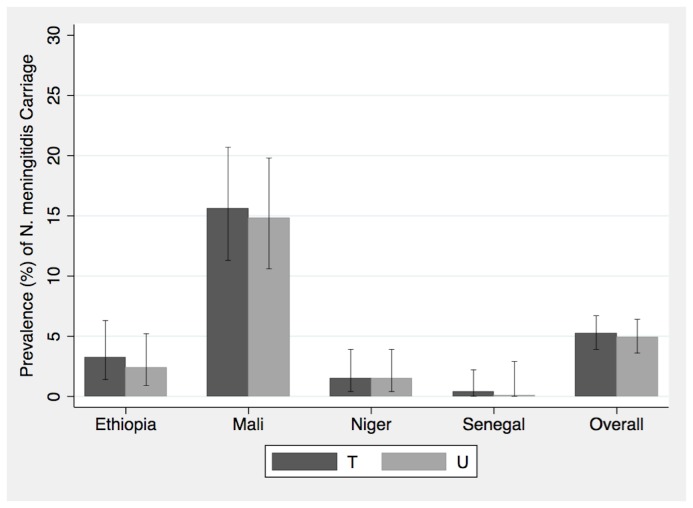
Comparison of *N. meningitidis* carriage by swabbing method. The prevalence and 95% confidence intervals of *N. meningitidis* carriage as determined by swabbing the posterior pharynx behind the uvula (U) or swabbing the posterior pharynx behind the uvula plus one tonsil (T) by center and overall.

Prevalence of *N. meningitidis* carriage from the T swab was 5.2% (95% CI: 3.9-6.7%) compared with a prevalence of 4.9% (95% CI: 3.6-6.4%) from the U swab (McNemar’s Test for paired samples, p= 0.6) (Table 2). Concordance between the two methods was 96.3% and the *kappa* was 0.61 (95% CI 0.50-0.73), indicating good agreement beyond chance between the two methods. However in 2.0% (95% CI: 1.2-3.1%) of children only the T swab was positive for carriage, and in 1.7% (95% CI: 1.0-2.7%) only the U swab was positive. Both swabs were positive in 3.2% (95% CI: 2.2-4.5%). Prevalence of carriage was higher in Mali than the other countries (Figure 1, supporting material Table S1). 

**Table 2:** Overall concordance between the two swabbing methods.

***PairedPharyngealSwabSamplesfromAllParticipants***		**T Method**		
		Positive	Negative	**Total**
**U Method**	Positive	32 (3.2%)	17 (1.7%)	**49 (4.9%)**
	Negative	20 (2.0%)	937 (93.1%)	**957 (95.1%)**
	**Total**	**52 (5.2%)**	**954 (94.8%)**	**1006**

Comparison of swabbing the posterior pharynx behind the uvula (U) or swabbing the posterior pharynx behind the uvula plus one tonsil (T) to determine carrier status.

Prevalence of carriage based on the first swab, regardless of the collection method, was 5.0% (95% CI: 3.7-6.5%) compared with prevalence from the second swab of 5.1% (95%CI: 3.8-6.6%) (McNemar’s Test for paired samples, p=0.9). Twenty five per cent of swabs collected were ordered T then U. Of the 50 first swabs that were positive, 44% were T swabs and 56% were U swabs. Of the 51 second swabs that were positive, 59% were T swabs and 41% were U swabs. While 50 children were identified as carriers based on the first swab, an additional 19 children (an additional 38%) were identified as carriers based on the result of the second swab. 

## Discussion

In our direct comparison of the two most commonly employed methods for collecting pharyngeal swabs to identify *N. meningitidis* carriers, we found that swabbing the posterior pharynx behind the uvula and one tonsil or swabbing the posterior pharynx alone provided similar estimates of the prevalence of carriage. Furthermore, our analysis demonstrates that the collection of two swabs regardless of method identified a higher proportion of carriers than collecting a single swab. Since both of the swabbing methods underestimated the prevalence of carriage, misclassification of carrier status is likely a concern in carriage studies regardless of which method of sampling is used. Our results suggest that future studies could minimize the potential for misclassification by collecting two swabs. 

Previous studies have shown that though meningococcal bacteria live on the surface of the tonsils and can be cultured from swabbing the surface, swabbing alone underestimates the true prevalence because the bacteria can also reside below the surface of the mucous membranes [21]. Previous research has also shown that swabbing behind the uvula alone identifies carriers more often that swabbing the tonsils alone [17]. Our results are consistent with this finding because both of the swabbing methods compared here included swabbing the posterior pharynx behind the uvula. In a previous study in the United Kingdom (UK), collecting two sequential swabs using the U method produced a very high level of concordance for carrier status (98%) [22]. Comparing sequential U swabs in the absence of a true gold standard means that the concordance can remain high even if the sensitivity of the screening method is low. However the increase in the observed prevalence following the second swab was minimal in the UK study compared to our study where a 38% increase in yield was observed. A limitation of the present study is that we did not obtain two consecutive swabs from the same site, although both swabbing methods included sampling from the posterior pharynx. Although our results suggest that future studies could minimize the potential for misclassification by collecting two swabs, the cost implications of double swabbing for prevalence studies would be significant and the extent of added benefit is worthy of further study. An analysis of the epidemiological aspects of these data including the age-specific prevalence, evaluation of risk factors for carriage in schoolchildren, and serogroups of the meningococci identified is underway and will be the focus of future work.

The specificity of either method for detecting carriers is likely to be high because the probability of identifying *N. meningitidis* following a multi-stage analysis that includes both culture and molecular tests in the absence of the bacterium is very low. While statistical methods to determine the specificity and sensitivity of two tests in the absence of a gold standard have been developed [23-26], these methods could not be applied because they rely upon the assumption that the two methods are independent, require knowledge about at least one of the tests, need prior information about the prevalence of the condition in the population, or require more than two tests. 

The prevalence of meningococcal carriage in this pilot study varied considerably between centers. We concluded at the time that the very low carriage observed in most centers reflected a need to strengthen microbiological methods before the main surveys. However, given that this analysis is based on paired samples, any variation in observed overall carriage across the centers should not affect the comparison of the two swabbing methods. Indeed, the relative prevalence based on the two swabbing methods were similar across the centers. 

The first documented *N. meningitidis* carriage prevalence study in Africa was conducted in 1915 among British soldiers stationed in Sudan [4,27], but researchers have long recognized that the reliability of the results of early studies may be questionable due to the difficulty of culturing, isolating, and identifying the bacteria, leading to misclassification [4]. Yet, nearly a century later, no gold standard for the collection and analysis of swab samples for identifying meningococcal carriers has been established. A true gold standard is needed to evaluate the sensitivity and specificity of different methods, to monitor newly emerging phenotypes and genotypes, to implement epidemiological studies to assess risk factors and the impact of vaccines on carriage, and to better understand the relationship between carriage and immunity. Methods that maximize the sensitivity of detecting carriers would be most informative in this regard. Techniques such as polymerase chain reaction (PCR) for directly identifying *N. meningitis* from the swab could improve sensitivity by eliminating the need for culture-based methods, and are worthy of further investigation. 

## Supporting Information

Table S1 **Center-specific concordance between the two swabbing methods.** Comparison of swabbing the posterior pharynx behind the uvula (U) or swabbing the posterior pharynx behind the uvula plus one tonsil (T) to determine carrier status by center.
(DOCX)
