# Supplementary material for: Methods for Identifying Neisseria meningitidis Carriers: A Multi-Center Study in the African Meningitis Belt
Source: PLoS One. 2013 Oct 23;8(10):e78336. doi: 10.1371/journal.pone.0078336 (PMC3806823; doi:10.1371/journal.pone.0078336)
Supplement: Table S1 — Center-specific concordance between the two swabbing methods. Comparison of swabbing the posterior pharynx behind the uvula (U) or swabbing the posterior pharynx behind the uvula plus one tonsil (T) to determine carrier status by center. (DOCX) [file pone.0078336.s001.docx]

***Table S1: Center-specific concordance between the two swabbing methods.*** Comparison of swabbing the posterior pharynx behind the uvula (U) or swabbing the posterior pharynx behind the uvula plus one tonsil (T) to determine carrier status by center.

| ***Table S1a:*** ***Ethiopia –*** | |  | |  |
| --- | --- | --- | --- | --- |
| ***Paired Pharyngeal Swab Samples*** | | **T Method** | |  |
|  | | Positive | Negative | **Total** |
| **U Method** | Positive | 0 | 6 (2.4%) | **6 (2.4%)** |
|  | Negative | 8 (3.2%) | 234 (94.4%) | **242 (97.6%)** |
|  | **Total** | **8 (3.2%)** | **240 (96.8%)** | **248** |

| ***Table S1b:*** ***Mali –*** | |  | |  |
| --- | --- | --- | --- | --- |
| ***Paired Pharyngeal Swab Samples*** | | **T Method** | |  |
|  | | Positive | Negative | **Total** |
| **U Method** | Positive | 28 (11.6%) | 9 (3.6%) | **37 (14.8%)** |
|  | Negative | 11 (4.4%) | 202 (80.8%) | **213 (85.2%)** |
|  | **Total** | **39 (15.6%)** | **211 (84.4%)** | **250** |

| ***Table S1c:*** ***Niger –*** | |  | |  |
| --- | --- | --- | --- | --- |
| ***Paired Pharyngeal Swab Samples*** | | **T Method** | |  |
|  | | Positive | Negative | **Total** |
| **U Method** | Positive | 4 (1.5%) | 0 | **4 (1.5%)** |
|  | Negative | 0 | 258 (98.5%) | **258 (98.5%)** |
|  | **Total** | **4 (1.5%)** | **258 (98.5%)** | **262** |

| ***Table S1d: Senegal –*** | | ***T Method*** | |  |
| --- | --- | --- | --- | --- |
| ***Paired Pharyngeal Swab Samples*** | |  | |  |
|  | | Positive | Negative | **Total** |
| **U Method** | Positive | 0 | 2 (0.8%) | **2 (0.8%)** |
|  | Negative | 1 (0.4%) | 243 (98.7%) | **244 (99.2%)** |
|  | **Total** | **1 (0.4%)** | **245 (99.6%)** | **246** |
